# Autonomy and couples’ joint decision-making in healthcare

**DOI:** 10.1186/s12910-017-0241-6

**Published:** 2018-01-11

**Authors:** Pauline E. Osamor, Christine Grady

**Affiliations:** Department of Bioethics, Clinical Center, Building, 10/1C118, National Institutes of Health, Bethesda, MD 20892-1156 USA

**Keywords:** Autonomy, Couples, Joint decision-making, Healthcare

## Abstract

**Background:**

Respect for autonomy is a key principle in bioethics. However, respecting autonomy in practice is complex because most people define themselves and make decisions influenced by a complex network of social relationships. The extent to which individual autonomy operates for each partner within the context of decision-making within marital or similar relationships is largely unexplored. This paper explores issues related to decision-making by couples (couples’ joint decision-making) for health care and the circumstances under which such a practice should be respected as compatible with autonomous decision-making.

**Discussion:**

We discuss the concept of autonomy as it applies to persons and to actions, human interdependency and gender roles in decision-making, the dynamics and outcomes of couples’ joint decision-making, and the ethics of couples’ joint decision-making. We believe that the extent to which couples’ joint decision-making might be deemed *ethically* acceptable will vary depending on the context. Given that in many traditional marriages the woman is the less dominant partner, we consider a spectrum of scenarios of couples’ joint decision-making about a woman’s own health care that move from those that are acceptably autonomous to those that are not consistent with respecting the woman’s autonomous decision-making. To the extent that there is evidence that both members of a couple understand a decision, intend it, and that neither completely controls the other, couples’ joint decision-making should be viewed as consistent with the principle of respect for the woman’s autonomy. At the other end of the spectrum are decisions made by the man without the woman’s input, representing domination of one partner by the other.

**Conclusions:**

We recommend viewing the dynamics of couples’ joint decision-making as existing on a continuum of degrees of autonomy. This continuum-based perspective implies that couples’ joint decision-making should not be taken at face value but should be assessed against the specific cultural, ethnic, and religious backgrounds and personal circumstances of the individuals in question.

## Background

A prevalent and widely accepted perspective in bioethics is that competent adults have a right to make their own decisions about whether to seek health care, receive healthcare interventions, or participate in clinical research. This is consistent with the principle of respect for autonomy, which entails respect for persons’ capacity to be self-determining and their right to direct their own lives. Respecting autonomy in practice, however, is complex since most persons define themselves and make decisions influenced by a complex network of social relationships. Respecting their autonomy, therefore, may involve understanding and respecting relationships that are important to them and the process with which they incorporate the values inherent in these relationships into their decision-making.

One of the most common social relationships in which decision-making takes place is that of couples/partners. Married or cohabiting couples make decisions together in several domains of life, often including health care. The dynamics of this decision-making process for each individual in the couple has not been well studied. In a recent analysis of Nigerian women’s responses to a question about who makes healthcare decisions for them, a third of the women reported that decisions about their healthcare were made by them and their husbands/partners jointly, while only 6% of women reported making healthcare decisions for themselves and 61% reported that their husbands/partners make decisions for them [1]. While it is apparent that healthcare decisions made for the woman by her husband/partner alone without her participation represents a lack of decision-making autonomy for the woman, joint decision-making may or may not represent a level of partnership or equality within the couple that is consistent with the woman’s decision-making autonomy.

The concept of couples’ joint decision-making and how it coheres with autonomous decision-making deserves additional study. Intuitively, joint decision-making seems to be a promising strategy for many real-life situations in which people find themselves. For instance, it may be preferable for a couple to be involved in joint decision-making because many issues affecting one member of the couple affects them both, just as it does when two parents make decisions about their child. Our focus in this paper is on couples’ joint decision-making, particularly whether and when it should count as acceptably close to autonomous decision-making. Given that in traditional male-female relationships, women are often the less dominant partner in decision-making [2–6], we explore the issue of couples’ joint decision-making in the context of women’s healthcare decisions. While we acknowledge that couples consist of various gender combinations and that this analysis may be applicable for all couples regardless of gender or who is the dominant member, in this paper our focus is on traditional marriage (or similar unions) between a man and woman. In this paper, we explore the notion of couples’ joint decision-making, asking under what circumstances such a practice should be respected as compatible with autonomous decision-making. After considering theories of autonomy and data on joint decision-making and its association with healthcare outcomes, gender roles, and community norms in healthcare decisions, we consider a spectrum of scenarios of couples’ joint decision-making about a woman’s own health care that move from those that are acceptably autonomous to those that are less or not consistent with respecting the woman’s autonomous decision-making.

### The nature of autonomy as a principle in bioethics

Respect for autonomy is one of the guiding principles of bioethics. Respect for autonomy might entail respect for an autonomous person or respect for an autonomous action or decision [7]. An autonomous person is one capable of self-legislation and able to make judgments and take actions based on his/her particular set of values, preferences, and beliefs [8]. Many theories of autonomy describe two necessary conditions for a person to be autonomous: liberty (freedom from controlling influences) and agency (capacity for intentional action) [9, 10]. To respect an autonomous agent is to acknowledge that person’s right to hold views, to make choices and take action based on his or her own personal values and beliefs [11]. Most adults are capable of being autonomous however, persons otherwise capable of being autonomous sometimes do not make autonomous decisions. Beauchamp and Childress [11] describe three qualities an agent must possess in relation to a specific choice for that choice to be autonomous: 1) intentionality: the agent intends to perform the action, 2) understanding: the agent understands the action he or she is choosing, and 3) no external control: the agent is not controlled by another.

Intentional acts require a plan of action, although not necessarily reflective thought or strategy. We do many things intentionally but without thought, such as reaching for a glass of water in order to drink or flipping a page in a book. The condition of intentionality is explained in terms of a plan for the achievement of an end guided by values and preferences, and not just an intended end [11]. For example, a woman with breast cancer who wants to get better has options for treatment ranging from faith healing to invasive surgery. If she does not plan for or decide on treatment to achieve her end, then she has not satisfied the condition of intentionality. One need not have a perfect understanding of the relevant information in order to make an autonomous decision or act autonomously, but “substantial understanding,” taken as full or perfect understanding is a prohibitively high threshold. A woman facing the prospect of mastectomy for breast cancer, for example, should understand that her breasts will be surgically removed but does not need to understand the specific surgical procedure the surgeon will use. Many conditions, such as youth, mental illness, inattention, unfamiliarity, or insufficient information given by medical professionals can result in inadequate understanding [11]. Sufficient freedom from external control from another allows a person to act or choose autonomously if she intends and understands the action. A person can be influenced without being controlled by another. By contrast, completely controlled acts are entirely dominated by the will of another. Not all influences are controlling; certain influences can facilitate choice and are welcomed by the persons on whom they operate. A woman who post-mastectomy for breast cancer has breast reconstruction because her partner tells her to without consideration of her wishes is dominated by him. Another woman in the same position may welcome support and input from a partner who respects her autonomy and helps her decide, but she makes the final decision. Fasse et al. [12] in their study revealed that the decision-making process among couples about reconstruction surgery after breast cancer was described as an interrelated experience in which the husband/partner’s role was consultative and mostly supportive. Indeed, the second woman above may come to the same decision as the first woman but the role of her partner is not control.

Whether or not a choice is sufficiently uncontrolled to be considered autonomous depends at least in part on the kind and degree of influence placed on the chooser. Three types of influence are often distinguished: coercion, manipulation and persuasion. Coercion “occurs only if an intended and credible threat displaces a person’s self-directed course of action, thereby rendering even intentional and well informed behavior non-autonomous.” A woman who chooses breast reconstruction surgery because her partner threatens to leave her if she does not is coerced into making that choice. Because it illegitimately controls another person’s decision and usurps autonomy, coercion is wrong. Persuasion, in contrast, is a non-controlling (resistible) form of influence which respects autonomy. By persuasion, one person intentionally and successfully uses reasons and facts to convince another person to willingly pursue an action. For example, a woman’s partner may persuade her to choose breast reconstruction because he knows how much her appearance means to her, and that she will feel more at ease about the way her body looks if she undergoes reconstruction. Between persuasion and coercion lies a group of influential behaviors included under the broad definition of manipulation. Faden and Beauchamp define manipulation as a “catch-all category for any intentional and successful influence of a person by non-coercively altering the *actual choices* available to the person or non-persuasively altering the *person’s perceptions of those choices,*” (italics ours- p.236 of [13]), a distinction utilized by others [14]. For example, manipulation (including instilling fear or skepticism about modern medicine) might be used to get a patient to refuse life-saving treatment (such as blood transfusion or surgery) or a mother to refuse vaccination for her children. Manipulation is usually wrong because it disrespects autonomy, however, it may not always invalidate autonomous choice if it is sufficiently non-controlling in situations where intentionality and understanding are intact [14]. Continuing with the example above, a woman might be manipulated into choosing breast reconstruction if her partner limits her range or perception of options by implying that she may be less attractive to him without reconstruction, limiting what he is willing to help pay for, or convincing her that surgery is unacceptably onerous. Her decision may still be sufficiently autonomous, however, if she has adequate understanding about the range of options and knows she can choose something other than what her partner wants.

Typically, decisions are made in a context of competing influences such as familial constraints, legal obligations and institutional pressures, influences that usually do not control decisions to a morally worrisome degree. Controlling influences (including coercion, undue pressuring, and some types of manipulation) render an action non-autonomous because it is not voluntary, while non-controlling influences do not destroy the voluntariness of a person’s decisions. Although respect for autonomy is an important and perhaps universal principle, a conception of autonomy that overemphasizes reliance on individuals being independent and self-sufficient can distort understanding of the way individual decisions are embedded in a web of relationships and familial values [15]. Acknowledging cultural variation is also necessary for evaluating the relevance and applicability of international ethical principles [16]. Indeed, it may not be possible to apply general principles of autonomy without recognizing the relevance of social networks, detailed local knowledge and cultural norms.

### Human interdependency and the complexity of decision-making

Feminists and others have sought to revise atomistic conceptions of autonomy to include ideas of “relational autonomy” [17]. Relational autonomy is based on the understanding that persons are socially embedded and that their identities are formed within the context of social relationships and shaped by a complex of intersecting social determinants, such as race, class, gender, and ethnicity. The concept of relational autonomy seeks to balance notions of independent autonomous agency with the reality of social embeddedness [17, 18]. Feminist thinkers highlight that insofar as autonomy is dependent on agency, agency is always exercised by an embedded self, and so to think about the autonomous decision-maker as an isolated individual is confusing. Since “others” will always be part of the exercise of one’s agency in some form or other, interdependence should be recognized as the norm rather than independence.

Interdependence refers to the effects interacting persons have on each other [19]. Most persons are interconnected and in relationships, and acquire habits of action and thought within social circumstances [20]. It is expected that these habits of action and thought include (among others) how people perceive and act in situations requiring healthcare decision-making. Most people around the world experience their lives in the context of relationships, including family, community, cultural groups and tribes. Decision-making in general, including healthcare decision-making occurs within the framework of these relationships.

### Gender roles and community norms in healthcare decision-making

Gender represents a complex interrelationship between an individual’s biological sex, internal sense of self and outward presentations and behaviors and is influenced by the roles, rights, and obligations attached by society to individuals designated at birth as either male or female. In many countries in the world, inequalities exist between genders such that men have and can exercise greater power than women in most spheres of functioning, men also have culturally and often legally sanctioned power over women and greater control of and access to resources and information. Thus, women‘s position in society and the degree to which they can exercise autonomy are often defined and limited by gender roles and gender relations in their societies and cultures.

In traditional societies, social, cultural, and, in some cases, legal constructs and practices contribute to low decision-making authority of women. Women’s autonomy is constrained by gender stratification and patriarchal authority and they often have considerably lower social status and autonomy than men [2, 3, 21, 22]. Evidence from Nepal, as in most parts of South Asia and from Nigeria, as in most parts of West Africa, show that women commonly have less power and autonomy than men in making decisions about their own health care [1, 4–6]. In many places, men have more power and the final say in decisions, the ability to make decisions without consulting another, or the freedom to make decisions without repercussions from another person. In predominantly patriarchal societies that emphasize women’s dependence on male kin, culturally appropriate behavior for women does not encourage expressions of individual autonomy or decision-making. In such societies, when a decision concerning a woman is made after discussion within the couple, it may not be apparent whether the decision was made jointly or if the woman is just doing what she has been told by her husband/partner. Thus, it is important to consider what women mean by any expression of couples’ joint decision-making, especially against the background, cultural and religious norms of the specific society in which a woman claiming joint decision-making lives.

### Couples’ joint decision-making

Joint decision-making within a couple is usually a critical part of family life [23, 24]. Couples’ decision-making is conceptualized as dynamic and interactive, occurring in the context of the marital or intimate relationship. If both couple partners participate in a decision that affects either of them, a better outcome may result than if either member alone decides, simply because it is likely that more options are explored when each partner voices his or her perspective [25–27]. Deciding jointly may also have the advantage of allowing each partner to express respect and care for the other partner, and take into account how decisions about one may affect the other. Despite its apparent prominence, couples’ joint decision-making has remained a relatively under-researched area in health care. Most studies of couples focused on other aspects of relationship dynamics, such as the amount and style of communication between the couple [12, 28], husband-wife power interactions [29] and couples’ perceptions of social support based on everyday life experiences of sharing household burdens [30]. Studies that have focused on decision-making in relationships have usually been in the context of who has the final say in decision-making [31, 32].

Studies on healthcare joint decision-making have not focused on couples, but rather on medical decision-making by surrogates [33, 34], shared medical decision-making between patient and healthcare provider [35], parents making medical decisions for children [36] and end-of-life decisions [37]. The few existing couple studies have examined older couples’ decision-making on health issues as it relates to health promotion behavior [38], couples’ household decision-making dynamics [25] and the association between various health outcomes and husband/partner involvement in the woman’s reproductive healthcare decisions [26, 39, 40].

### Couple joint decision-making and health outcomes

Empirical studies show that how decisions are made, and by whom, can have a profound effect on health status and outcomes. Studies on the association between various maternal and reproductive healthcare outcomes and couples’ household decision-making dynamics [25, 26, 40] use decision-making as a proxy measure of either women’s relative power [25, 26], or women’s autonomy [40]. In Sub-Saharan Africa, for example, women’s ability to make decisions in consultation with other family members is an important determinant of their access to and use of skilled maternal health services [41]. Data support the notion that couple’s joint decision-making is more favorable for maternal health outcomes than decisions made by only one partner [25].

Other studies found that joint decision-making and couple communication is associated with increased contraceptive use [42, 43], increased use of HIV related health-enhancing behaviors [44] and decreased risks of interpersonal violence [45]. Couples’ joint decision-making may also yield better reproductive health outcomes than when men make these decisions alone or women make decisions devoid of input from significant others [46]. Therefore, there are apparent pragmatic advantages of couples’ joint decision-making beyond respect for women’s autonomy. In describing decision-making patterns, some women report that joint decision-making is most desirable, because it allows a husband and wife to ‘share the blame’ in case negative repercussions ensue after a decision [46]. This concept highlights the importance of respecting women’s values and preferences when we care about autonomy. As Carter [47] pointed out, female autonomy, when thought of as the woman making individual decisions in isolation of her partner may not represent the ‘ideal’ in women’s eyes. In certain contexts, a woman making decisions alone implies that, rather than being empowered to make decisions, she is in fact bearing the burden of full responsibility and potential blame for those decisions.

### The ethics of couple joint decision-making

**Fig. 1:**
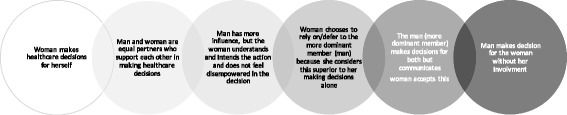
Continuum of the ethics of couples joint decision decision making for women’s health care

Based on understanding autonomous decisions and its components as distinct from the capacity to be an autonomous agent, the social embeddedness and interdependence of persons, gender and authority structures, and the limited literature on joint decision-making especially by couples, we propose a continuum of possible scenarios illustrating the ethical acceptability of couple’s joint decision-making for a woman’s healthcare. We propose that healthcare decisions for the woman made by a couple are acceptably autonomous when the three features of autonomous action and decision-making (intentionality, understanding and lack of external control) are sufficiently present in a couple *as a couple,* without one person’s preferences being imposed on the other or one person being controlled by the other person. Whether couples’ joint decision-making is deemed ethically acceptable is likely to vary depending on the context. To the extent that there is evidence that both members of the couple understand the decision, intend it, and that neither is completely controlled by the other, couples’ joint decision-making is acceptable. We propose that these features exist on a continuum of joint decision-making, where at one end, the woman’s’ participation in the decision is essentially equivalent to autonomous decision-making with support. At the other end, the woman’s decision is controlled and not consistent with respecting her autonomy. However, even at the less autonomous end of the continuum, involving the woman in discussions is preferable to not involving her at all, and for certain decisions this arrangement ought to be respected (Fig. 1).

1. *Couples’ joint decision-making should count as acceptably autonomous decision-making because it is equivalent to the woman making independent decisions.* In this scenario, the two people in the relationship are equal partners (neither partner is dominant over the other), both sufficiently understand the decision and intend to make it, they share decisions and support each other, such that making-decisions jointly supports the autonomous decision-making of each.
2. *Couples’ joint decision-making could count as acceptably autonomous decision-making even though individual decision-making (*e.g. *the woman making independent decisions) might be more autonomous.* In this scenario, it is acknowledged that, in real life, there are many relationships in which one partner has more power or holds more sway than the other depending on the issue being addressed. However, such couples might still make decisions jointly that are ethically acceptable as long as the less dominant partner has sufficient understanding and intentionality and does not view herself as disempowered. Indeed, one can autonomously rely on or defer to someone else when making a particular decision. Therefore, the fact that this exercise of autonomy may seem less than the theoretical ideal of individual autonomy, it does not render one partner not autonomous, and should be ethically acceptable
3. *Couples’ joint decision-making could count as acceptably autonomous decision-making when it better represents the values and preferences of women in certain societies.* In some traditional societies, a woman making individual decisions in isolation of her husband/partner may not see herself as making a more autonomous decision than if she was engaged in joint-decision-making with her spouse. She may consider individual decision-making inferior to joint decision-making. At the heart of this notion is that the autonomous preferences of a woman to engage her husband/partner and rely on him for decision-making should be respected from the perspective of the woman and respect for *her understanding* of what is acceptably autonomous in decision making and respect for her autonomous preferences.
4. *Couples’ joint decision-making does not meet the standards of acceptably autonomous decision-making but may be still ethically preferable to the alternative of the male partner making healthcare decisions for a woman without involving her at all.* In this scenario, couples’ “joint decision-making” falls short of recognizing the woman’s autonomy in making the decision. Nonetheless, since it still involves her in some way in the decision, it is preferable to not involving her at all, and may in certain circumstances be an acceptable compromise that permits healthcare to proceed. For example, if a woman diagnosed with breast cancer has to decide among several options in a context where she is entirely dependent on her spouse, has no access to resources without her spouse’s support and has no way of obtaining the treatment she chooses if she makes the decision on her own without her spouse. Waiting for a better decision making process in this scenario may lead to a worse outcome for the woman. This option prioritizes achieving better health outcomes for women over respecting women’s autonomy.

Some might worry that, on the other hand, couples’ joint decision-making can *never* be truly comparable to a woman’s autonomous decision making and is therefore ethically problematic because it allows another person to influence an individual’s decision thereby possibly reducing her autonomy. This viewpoint fails to recognize that not all influence is controlling, as described earlier, and further fails to recognize that autonomous decision-making understood as the act of an independent, self-sufficient individual is seldom realized in real life.

Therefore, in assessing the ethical acceptability of joint decision making, we propose that it is essential to consider the underlying dynamics of couples’ joint decision- making on a continuum in terms of autonomy. At one end as shown in Fig. 1, couples’ joint decision-making may be indistinguishable from individual decision-making while at the other end it represents domination of one partner by the other. As one moves along the spectrum from left to right, the autonomy of the woman decreases. This approach implies that statements of couples’ joint decision- making should not be taken at face value but appraised in the specific cultural, ethnic, religious, and personal context and background of the individuals in question.

Clinical assessment of couples’ power and decision-making dynamics is challenging and not easy or straight-forward. Assessment will depend not only on the cultural and personal context as described above, but also on the nature and seriousness of the decision. Further discussion and empirical research are necessary to develop methods that can be used to evaluate couples’ joint decision-making in clinical settings. Such research could lead to the development of clinical tools and approaches that can help assess couples’ joint decision making, detect potential domination by one partner that may be unduly pushing decision making in one direction and/or evaluate the presence of power imbalances, and “hidden” power, and how these affect decisions that are made. These issues can be complex in all cases, but especially when treating women from non-Western cultures whose notions of autonomy and decision-making norms may differ from Western concepts and norms.

## Conclusion

The bioethical principle of respect for autonomy entails respect for the capacity of persons to be self-determining and their right to direct their own lives. In health care, we respect and honor the decisions of autonomous adults. Respecting autonomy in practice, however, is complex since most persons make decisions influenced by a complex network of social relationships. In this paper, we examined joint decision-making by a married or cohabitating couple, one of the most prevalent social relationships globally. Couples’ joint decision-making may reflect growing equality and partnership between men and women when viewed against historical and traditional norms of men having decision-making authority. On the other end of the continuum, couples’ joint decision-making could reflect pressure from a spouse to present a united front to outsiders when one partner (usually the man) is actually making the decisions and there is no evidence of any respect or involvement of the other partner (often the woman). This continuum-based perspective implies that couples’ joint decision-making should be assessed against the specific cultural, ethnic, and religious backgrounds of the individuals in question and the particulars of their relationship and the decision to be made. Such assessment in a clinical or healthcare context would help to determine if the woman receiving healthcare who claims she arrived at the decision jointly with her husband/partner had an ethically acceptable level of autonomy when making decisions. Additional attention and research are needed to expand our understandings of this continuum and to develop and test effective methods of clinical assessment.
